# The antihypertensive agent hydralazine reduced extracellular matrix synthesis and liver fibrosis in nonalcoholic steatohepatitis exacerbated by hypertension

**DOI:** 10.1371/journal.pone.0243846

**Published:** 2020-12-14

**Authors:** Yuan Yuan, Hisao Naito, Kazuya Kitamori, Sayuki Hashimoto, Tomomi Asano, Tamie Nakajima

**Affiliations:** 1 College of Life and Health Sciences, Chubu University, Kasugai, Aichi, Japan; 2 Department of Public Health, Fujita Health University School of Medicine, Toyoake, Aichi, Japan; 3 College of Human Life and Environment, Kinjo Gakuin University, Nagoya, Aichi, Japan; Policlinico Universitario Campus Bio-Medico, ITALY

## Abstract

Hypertension is an important risk factor for nonalcoholic steatohepatitis. We have previously demonstrated that hypertensive rats fed a high fat and cholesterol (HFC) diet incurred a more severe hepatic inflammatory response and fibrosis. Here we investigated the role of hypertension in NASH by comparing HFC-induced hepatic fibrogenesis between spontaneously hypertensive rats (SHRs) and their normotensive Wistar Kyoto counterpart. Compared to the counterpart, the HFC diet led to stronger aggregation of CD68-positive macrophages in SHRs. HFC feeding also resulted in significantly higher upregulation of the fibrosis-related gene alpha-smooth muscle actin in SHR. The HFC diet induced higher overexpression of serum tissue inhibitor of metalloproteinase-1 (TIMP1) and greater suppression of matrix metalloproteinase-2 (MMP2):TIMP1, MMP8:TIMP1, and MMP9:TIMP1 ratios, as a proxy of the activities of these MMPs in SHR. Administration of the antihypertensive agent hydralazine to SHRs significantly ameliorated HFC-induced liver fibrosis; it suppressed the aggregation of CD68-positive macrophages and the upregulation of platelet-derived growth factor receptor beta, and collagen, type 1, alpha-1 chain. In conclusion, a hypertensive environment exacerbated the hepatic fibrogenetic effects of the HFC diet; while the effects were partially reversed by the antihypertensive agent hydralazine. Our data suggest that antihypertensive drugs hold promise for treating NASH exacerbated by hypertension.

## Introduction

Nonalcoholic fatty liver disease (NAFLD) is one of the most prevalent chronic liver diseases worldwide. Its aggressive form, nonalcoholic steatohepatitis (NASH), can progress to cirrhosis and end-stage liver disease and consequently increase both morbidity and mortality [1]. Regarded as a hepatic manifestation of metabolic syndrome, NAFLD is frequently associated with hypertension and insulin resistance [2,3]. In particular, hypertension is an important risk factor for NAFLD. A higher incidence of NAFLD and NASH in patients with hypertension has been reported relative to the normotensive population [4,5]. Ikuta et al. showed that hypertension might enhance the progression of NASH in association with a reduction in the anti-oxidant capacity of the liver [6]. However, the relationship between hypertension and NAFLD is highly complex, and many aspects remain unclear.

Spontaneously hypertensive rats (SHRs) are a line of Wistar strain rats bred to be hypertensive [7]. The systolic blood pressures in the adult male SHR and Wistar Kyoto (WKY) rats, as the normotensive control for the former, are 235 and 130 mmHg, respectively. Our previous study showed that SHRs as well as rats of the stroke-prone spontaneously hypertensive5/Dmcr (SHRSP5/Dmcr) strain developed more severe steatohepatitis and hepatic fibrosis when fed a high fat and cholesterol (HFC) diet than did the normotensive WKY rats [8]. In a hypertensive milieu, the HFC diet induced greater activation of inflammatory signals [transforming growth factor-beta 1 (TGF-β1)/mitogen-activated protein kinase pathways (MAPK)] and suppressed anti-oxidative and anti-inflammatory [nuclear factor erythroid 2-related factor 2 pathway (Nrf2)] signals. Thus, increased hepatic inflammation in the hypertensive strains might be associated with worsened steatohepatitis induced by the HFC diet. Furthermore, hepatic CD68-expressing macrophages, which include Kupffer cells (liver-resident macrophages) and bone marrow-derived (recruited) macrophages, play essential roles in hepatic inflammation and fibrosis related to NASH due to their secretion of pro-inflammatory or profibrotic cytokines, such as tumor necrosis factor-alpha (TNF-α) and TGF-β [9]. In addition, chronic liver inflammation leads to further hepatic fibrosis characterized by excessive deposition of extracellular matrix (ECM) components; such deposition appears to be related to an imbalance in ECM synthesis and degradation mediated by HSCs [10,11]. HSCs are the major ECM-producing cells [12], whereas ECM proteolysis is regulated by MMPs, a group of zinc-dependent endopeptidases, and their inhibitor as well as the TIMP1, which is expressed by activated HSCs [13,14]. MMP2, MMP8, and MMP9 have all been reported to be associated with the progression of liver fibrosis and cirrhosis [15–17]. However, no investigation has been conducted on how hypertension influenced ECM synthesis and degradation in the progression of HFC-induced fibrosis.

Here, we first investigated the role of hypertension in fibrogenesis during the progression of NASH by comparing HFC-induced hepatic fibrosis between hypertensive SHR and normotensive WKY rats. We hypothesized that hypertension exacerbates hepatic fibrosis by aggravating HFC-induced imbalance between ECM synthesis and degradation, and the effect of hypertension on fibrogenesis can be reversed by antihypertensive therapy; to this end, we further investigated how antihypertensive therapy with hydralazine, a peripheral arterial vasodilator, ameliorated HFC-induced fibrogenesis in SHRs. The exacerbating effects of hypertension were demonstrated by our findings that HFC feeding resulted in increased aggregation of CD68-positive macrophages and higher overexpression of TIMP1 protein in hypertensive SHRs, which are involved in the regulation of ECM synthesis or degradation. Furthermore, antihypertensive therapy with hydralazine significantly ameliorated HFC-induced hepatic fibrosis in SHRs and partially reversed the effects of hypertension.

## Materials and methods

### Animals and experimental protocols

WKY/Izm and SHR/Izm rats used in the present study were purchased from Japan SLC, Inc. (Hamamatsu, Japan) and maintained according to the Guidelines for the Kinjo Gakuin University Animal Center and Animal Experiments at the Nagoya University Animal Center, respectively. All animal procedures were approved by the Experimental Animal Research Committee of Kinjo Gakuin University (No. 94 and 154) and The Animal Experiments Committee of Nagoya University Graduate School of Medicine (No. 24247)) Neither committee has members who belong to animal welfare experts on either animal experimental committee, however, the study was approved after being reviewed by the Institutional Animal Care and Use Committees of both universities. All of rats in each animal center were maintained under specific pathogen-free and were housed in a temperature and light-controlled environment (23 ± 2°C, 55 ± 5% humidity, 12-h light/dark cycle) with free access to the control diet and tap water. We observed health and well-being of animals by weighing them once a week.

**Fig 1:**
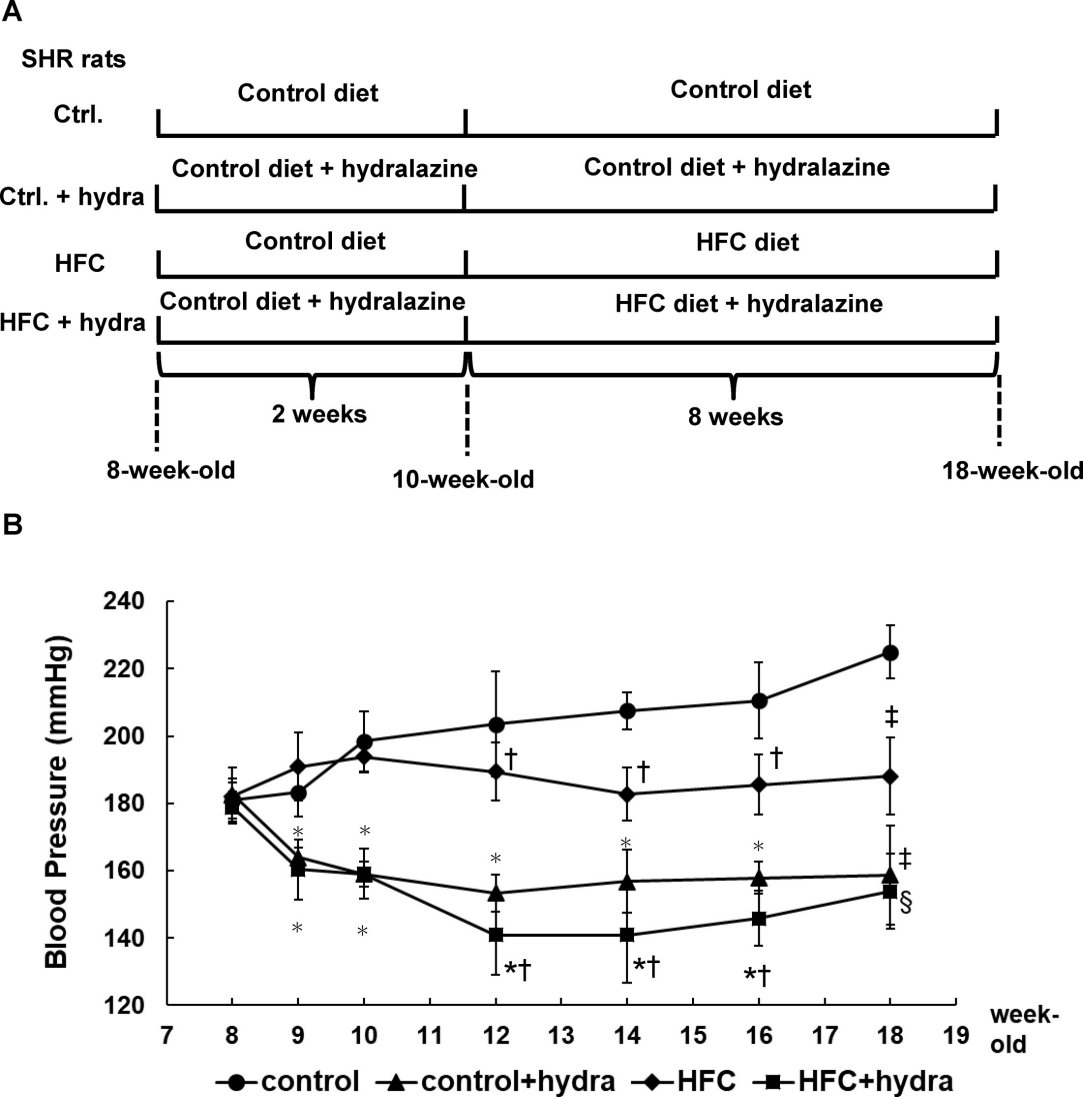
Experimental procedures and measurement of blood pressure. The experimental procedures (A). The 8-week-old male SHRs were randomly divided into 4 groups (*n* = 6) and fed control diet with or without hydralazine treatment (7.36 mg/kg body weight/day) for 2 weeks. Then they were fed HFC or control diet in the presence or absence of hydralazine for 8 weeks before the sacrifice. The effect of hydralazine on systolic blood pressure of SHRs (B). *P < 0.05 between control diet and HFC diet groups (two-way ANOVA); ^†^*P* < 0.05 between with and without hydralazine groups (two-way ANOVA); ^‡^*P* < 0.05 vs control diet group (one-way ANOVA); ^§^*P* < 0.05 vs control diet with hydralazine treatment (one-way ANOVA).

Two separate experiments were conducted. In the first experiment, male WKY or SHRs (8 weeks of age) were maintained on a control diet (Stroke-prone diet) for 2 weeks and then randomly divided into 2 groups (6 rats/group) and fed HFC (Stroke-prone diet supplemented with palm oil, cholesterol, and cholic acid) or control diets for 8 weeks, respectively. In the second experiment, 8-week-old male SHRs were divided into 4 groups (6 rats/group) and fed the control diet for 2 weeks, with or without the administration of hydralazine (7.42 mg/kg body weight/day in drinking water). They were then fed HFC or control diets for 8 weeks in the presence or absence of hydralazine (7.11–7.21 mg/kg body weight/day in drinking water), such that the groups comprised the following: 1) control diet, 2) control diet + hydralazine, 3) HFC diet, and 4) HFC diet + hydralazine (Fig 1). The detailed components of the HFC and control diets have been described previously [18].

At the end of the study duration, the first experimental group fasted overnight, while the second experimental group did not. All of the rats were anesthetized with pentobarbital (70 mg/kg); blood samples were collected and centrifuged at 3,500 ×g for 10 min for serum preparation. Then they were euthanized using an overdose of pentobarbital. The livers were removed, weighed individually, and then cut into pieces; one piece was fixed in 4% buffered paraformaldehyde; the remaining piece and corresponding serum sample were stored at −80°C until use.

### Real-time quantitative reverse transcription PCR (RT–qPCR)

Total RNA was extracted from the liver using an RNeasy Protect Mini Kit (QIAGEN, Tokyo, Japan). Complementary DNA was prepared from the total RNA (1μg) using Oligo (dT) 20 primer. RT–qPCR was performed with an Applied Biosystems 7900HT Fast Real-Time PCR System (Thermo Fisher Scientific, Waltham, MA), and expression was normalized to that of glyceraldehyde-3-phosphate dehydrogenase (GAPDH). The primers for collagen, type 1, alpha-1 chain (COL1A1) (Forward: ATGCTTGATCTGTATCTGCCACAAT; Reverse: ACTCGCCCTCCCGTTTTT; NM_053304) and GAPDH (Forward: AGAACATCATCCCTGCATCCA; Reverse: CCGTTCAGCTCTGGGATGAC; BC096440) were designed using their gene sequences in Primer Express (Applied Biosystems).

### Western blot analysis

Lysates were prepared by homogenizing liver pieces in 3 volumes of 0.25 M sucrose–10 mM phosphate buffer (pH 7.4), followed by centrifugation at 700 ×g for 10 min and collection of the supernatant. Western blotting was performed as described previously [8], using antibodies against alpha-smooth muscle actin (α-SMA) (ab5694, Abcam plc, Cambridge, UK) and platelet-derived growth factor receptor beta (PDGFR-β) (#3169, Cell Signaling Technology, Beverly, MA). Anti-glyceraldehyde-3-phosphate dehydrogenase (GAPDH) (sc-25778, Santa Cruz Biotechnology, Santa Cruz, CA, USA) was used for a loading control. Pierce 1-StepTM Ultra TMB-Blotting Solution (Pierce Biotechnology, Rockford, IL, USA) was used for signal development. Gray values for the target protein bands were measured with Image J (NIH, Maryland, USA) and normalized to the control group band value (defined as 1.0).

### Enzyme-linked immunosorbent assay (ELISA)

The serum levels of TNF-α, TGF-β1, TIMP1, MMP2, MMP8, and MMP9 were determined using Quantikine ELISA kits (R&D Systems, Minneapolis, MN) according to the manufacturer’s suggested protocols. Serum insulin was measured using a Morinaga Ultra Sensitive Rat Insulin ELISA kit (Morinaga Institute of Biological Science, Yokohama, Japan) per the manufacturer’s suggested protocols.

### Biochemical analyses

Serum triglycerides (TG), total cholesterol (TC), glucose, aspartate aminotransferase (AST), alanine aminotransferase (ALT), and gamma-glutamyl transferase (GGT) were measured by SRL Inc. (Tokyo, Japan). Hepatic lipids were extracted using the method of Folch et al. [19], as described previously [8], and hepatic TG and TC were evaluated using TG–IE and T–Cho IE kits (Wako, Osaka, Japan) per the manufacturer’s instructions, respectively.

### Histopathological and immunohistological analyses

The fixed liver samples (see Section 2.1.) were embedded in paraffin and sectioned at 4 μm. Hematoxylin and eosin (H&E) staining was performed to evaluate pathological conditions (e.g., hepatocyte degeneration (ballooning) and inflammatory cell infiltration), whereas Elastic Van Gieson (EVG) staining with Sirius red was conducted to determine the extent of fibrosis. The fibrotic areas in the EVG-stained sections were quantified in NIS-Elements (Nikon Instruments, Tokyo, Japan), as described previously [20].

Liver macrophages were identified via CD68 immunolabeling. Deparaffinized slides were pretreated with Histo/Zyme (Diagnostic BioSystems, Pleasanton, CA) for antigen retrieval and then blocked with H_2_O_2_ solution. Slides were incubated with anti-CD68 antibody at 4°C overnight, followed by incubation with MAX-PO (MULTI) secondary antibody (Nichirei Biosciences Inc., Tokyo, Japan). Finally, ImmPACT DAB Substrate (Vector Laboratories, Burlingame, CA) was used for color development.

### Measurement of blood pressure

To verify the antihypertensive effect of hydralazine, blood pressure was measured via tail-cuff and BP-98A blood-pressure gage (Softron, Tokyo, Japan) at the indicated time points (Fig 1).

### Thiobarbituric acid reactive substances (TBARS) assay

Lipid peroxidation and oxidative stress were assessed from serum samples using a TBARS assay kit (Cayman, Ann Arbor, MI, USA) in accordance with the manufacturer’s instructions.

### Statistical analysis

Data analysis was performed using Stata 15 statistical software package (Stata Crop, College Station, TX). All data were analyzed using two-way analysis of variance (ANOVA) between the strain and HFC diet or the HFC diet and hydralazine treatment. When the interaction was statistically significant, one-way ANOVA was performed to compare rats fed the HFC diet and those fed the control diet or with and without hydralazine treatment. The data were expressed as the mean ± standard deviation. To compare the effects of the HFC diet between SHRs and WKY rats, fold changes were calculated as the ratio of the HFC diet to control diet groups. A probability (*P*) value < 0.05 indicated statistical significance. Statistical analysis of non-normally distributed data was performed after log transformation of each value.

## Results

### CD68-positive macrophages

**Fig 2:**
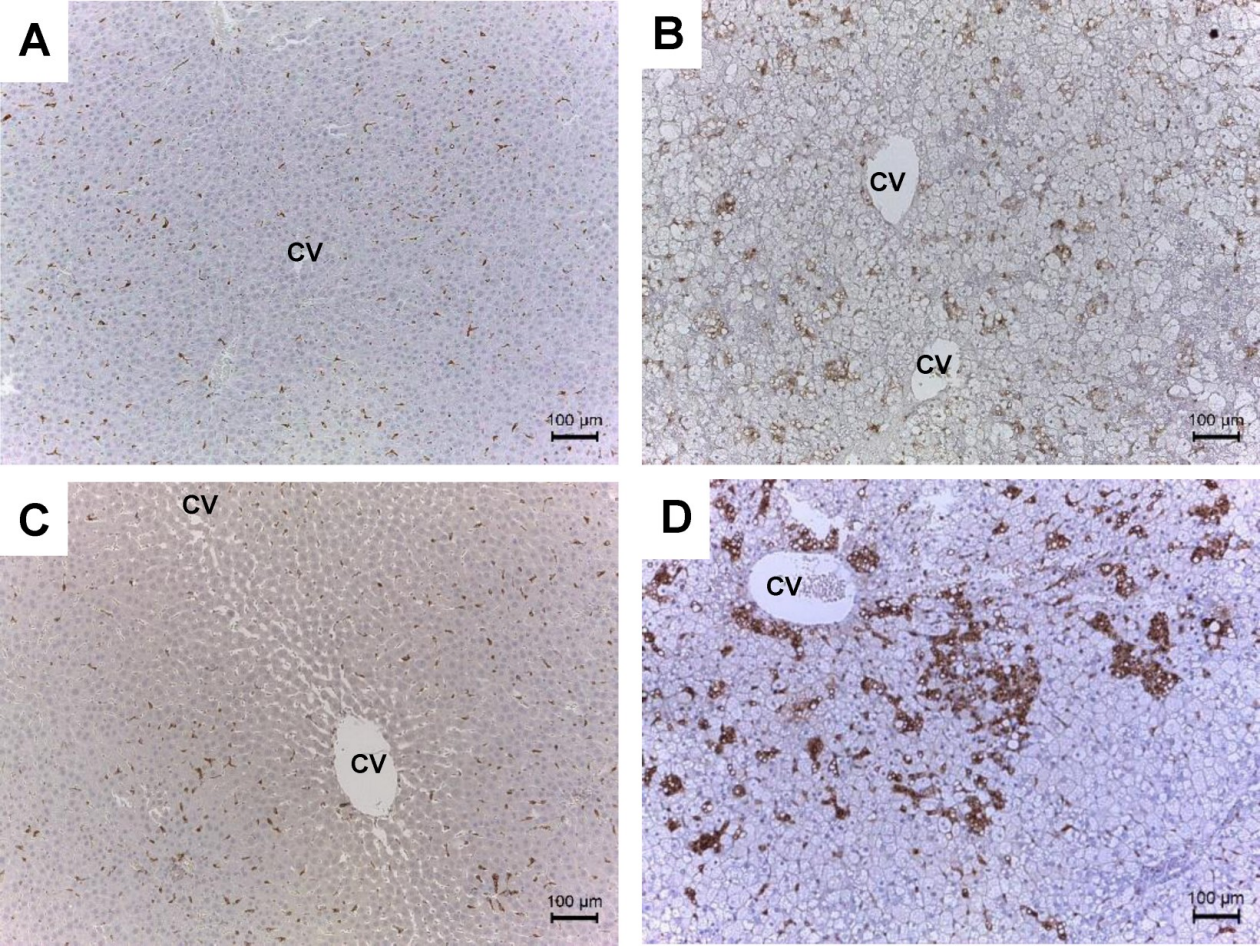
Images of liver sections in the first experiment. Representative images of liver sections subjected to immunostaining with CD68 antibody. Liver sections from normotensive WKY rats fed control (A) or HFC diet (B) and hypertensive SHRs fed control (C) or HFC diet (D) [magnification: ×100]. Scale bar, 100 μm. CV, central vein; CD68, cluster of differentiation 68.

Hepatic macrophage counts were compared between hypertensive SHRs and normotensive WKY rats fed the HFC or control diet. Scattered CD68-positive cells were observed in the livers of both WKY and SHRs fed the control diet (Fig 2A and 2C). The HFC diet induced moderate aggregation of CD68-positive cells in the livers of the WKY rats (Fig 2B), whereas the aggregation was more prominent in SHRs (Fig 2D).

### Fibrogenesis-related genes

**Fig 3:**
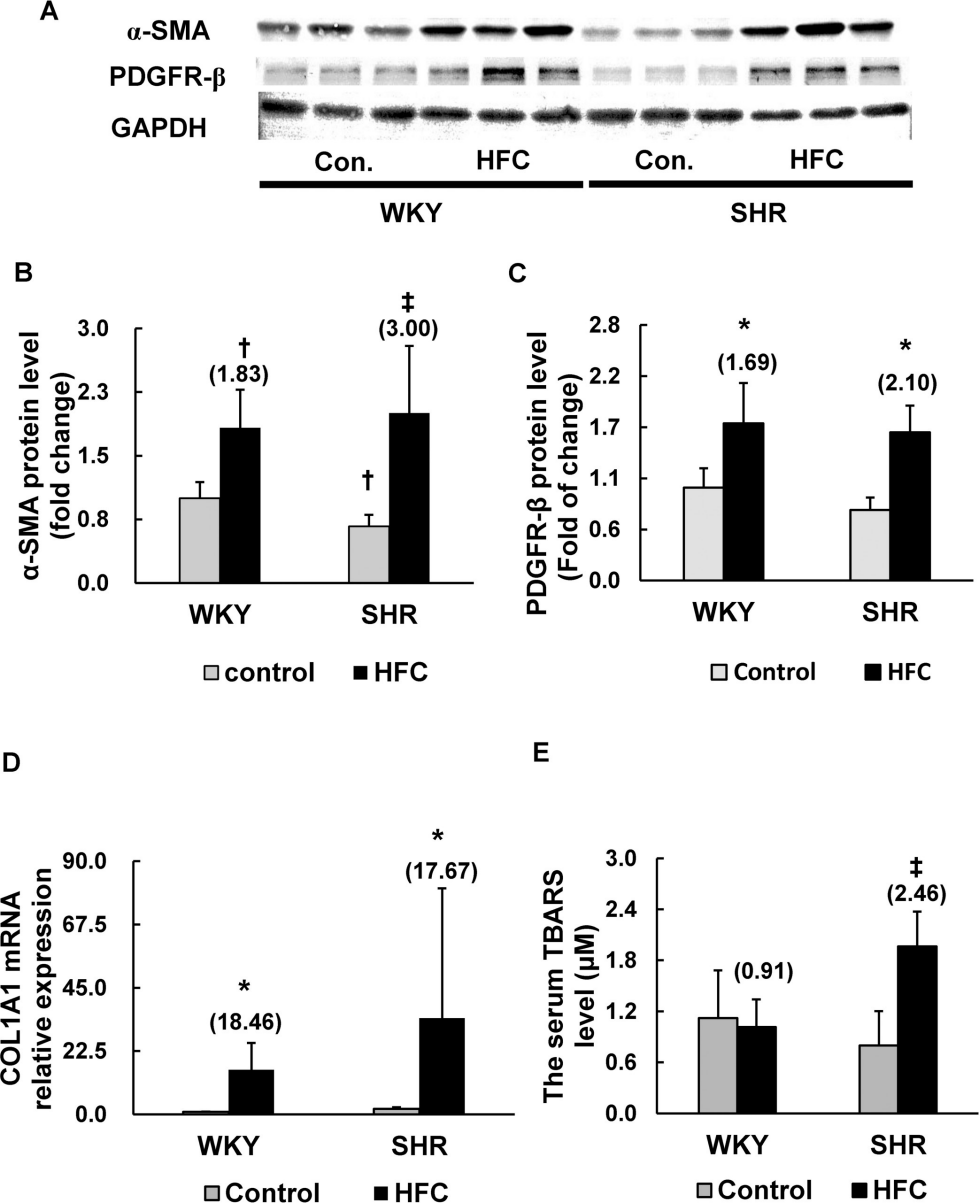
Effects of HFC diet on the expression of liver fibrosis-related genes. Western blots of the protein expression of α-SMA (A, B) and PDGFR-β (A, C) in the livers of WKY and SHRs fed the control or HFC diet. RT–qPCR measurements of the hepatic expression of COL1A1 mRNA (D). Serum TBARS levels representing levels of oxidative stress (E). *n* = 6/group. The values in parentheses represent the fold changes compared with the respective control. Significant interaction between the strain and HFC diet was observed in the hepatic levels of α-SMA and serum TBARS. *P < 0.05 vs respective control diet group with two-way analysis of variance (ANOVA); ^†^*P* < 0.05 vs WKY control diet group (one-way ANOVA); ^‡^*P* < 0.05 vs SHR control diet group (one-way ANOVA). α-SMA, alpha-smooth muscle actin; PDGFR-β, platelet-derived growth factor receptor-β; COL1A1, collagen, type I, alpha-1 chain; TBARS, 2-thiobarbituric acid reactive substances.

Activated HSCs expressed the fibrogenesis-related genes α-SMA and PDGFR-β, expressions of which were evaluated via western blotting (Fig 3A–3C). Furthermore, because the synthesis of collagen in HSCs has been suggested to be regulated at the transcriptional and posttranscriptional levels [14,21], the mRNA expression of COL1A1 was measured via RT–qPCR (Fig 3D). Significant interaction was observed in hepatic α-SMA levels between strain and HFC feeding but not in hepatic PDGFR-β protein and COL1A1 mRNA ones. The protein level of α-SMA, a myogenic marker, was slightly lower in the livers of SHRs fed the control diet relative to that in the WKY rats. The HFC diet significantly increased the expression of α-SMA and PDGFR-β proteins as well as COL1A1 mRNA in both WKY and SHRs. Notably, the increase in the level of α-SMA, in hypertensive SHRs (3.00-fold) was greater than that in the normotensive WKY strain (1.83-fold), suggesting that HFC-induced proliferation and activation of HSCs was more prominent in SHRs. However, the differences in HFC-induced elevation of PDGFR-β protein and COL1A1 mRNA were not observed between the 2 strains.

Because oxidative stress contributes to the progression of liver fibrosis [22,23], we also evaluated oxidative stress between SHRs and WKY rats by measuring serum levels of TBARS (Fig 3E). Significant interaction was observed in the serum levels of TBARS between the strain and HFC diet. The HFC diet had no effect on WKY rats, but it significantly increased the levels of TBARS in SHRs.

### MMPs/TIMP1

**Fig 4:**
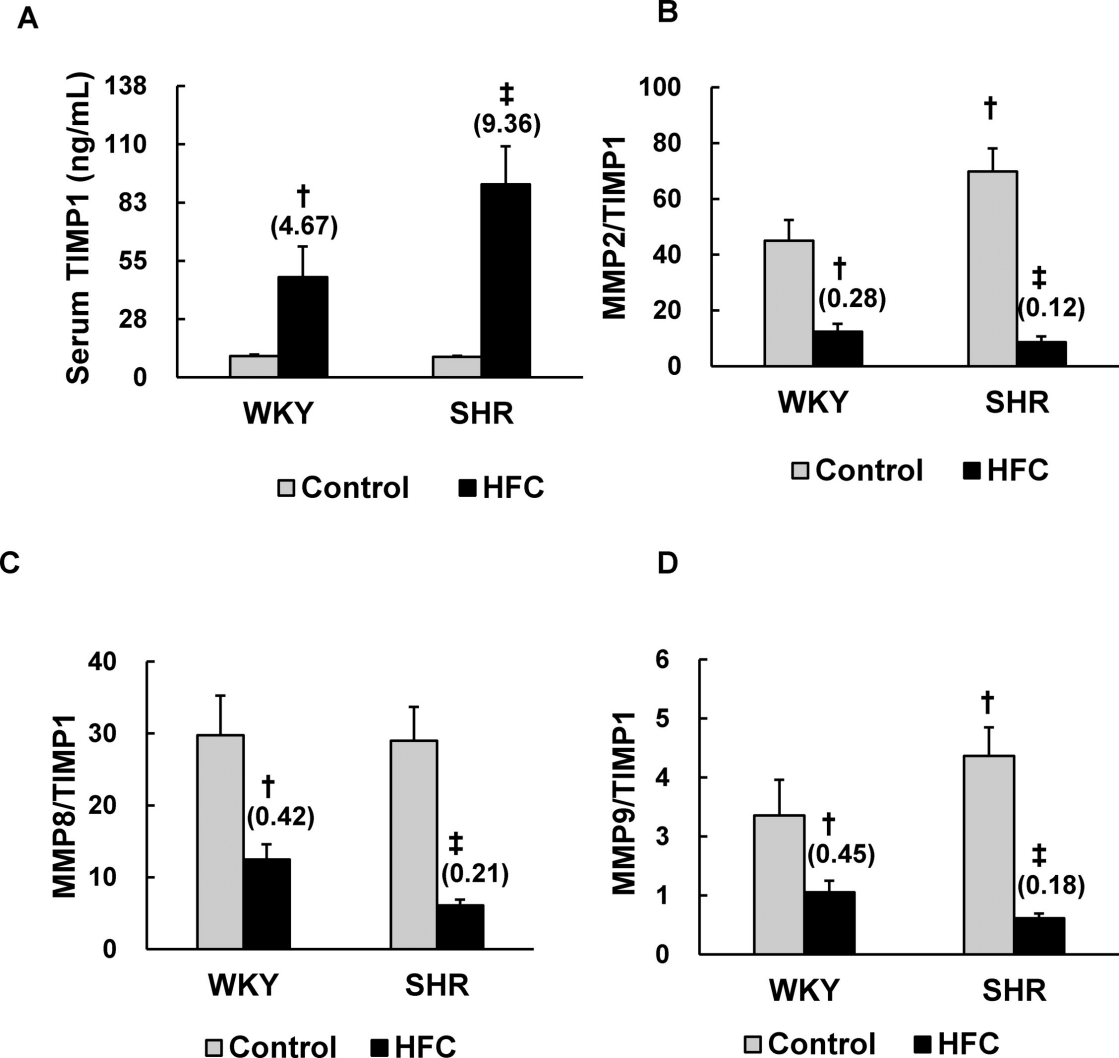
Effects of the HFC diet on serum MMPs/TIMP1. ELISA of serum TIMP1 (A) and MMPs (MMP2, MMP8, and MMP9) (B–D) in WKY and SHRs fed the control or HFC diet. The ratios of MMPs to TIMP1 were calculated as a proxy of MMP activity, n = 6/group. The values in parentheses represent the fold changes compared with the respective control. Significant interaction between stain and HFC diet was observed in serum TIMP1 and ratios of all MMPs to TIMP1. ^†^*P* < 0.05 vs WKY control diet group (one-way ANOVA); ^‡^*P* < 0.05 vs SHR control diet group (one-way ANOVA). TIMP1, tissue inhibitor of metalloproteinase; MMP, matrix metalloproteinases.

We evaluated the serum levels of MMP2, MMP8, MMP9, and their inhibitor TIMP1 via ELISA (Fig 4). Because TIMP1 inhibits MMP activity via binding the enzyme in a noncovalent 1:1 complex [24], the MMP:TIMP1 ratio was calculated as a proxy of MMP activity. Significant interaction between the strain and HFC diet was observed in the serum levels of TIMP1 and each MMPs:TIMP1 ratio. The HFC diet significantly elevated the level of serum TIMP1 in both WKY and SHRs; the increase observed in hypertensive SHRs (9.36-fold) was greater than that in the normotensive WKY rats (4.67-fold) (Fig 4A). The HFC diet induced greater decrease in MMP2:TIMP1 (0.12-fold), MMP8:TIMP1 (0.21-fold), and MMP9:TIMP1(0.18-fold) ratios in SHRs compared with those in the WKY strain (MMP2: 0.28-fold, MMP8: 0.42-fold, MMP9: 0.45-fold) (Fig 4B–4D), suggesting that HFC-induced inhibition of MMP activity was more prominent in hypertensive SHRs. Furthermore, the MMP2:TIMP1 and MMP9:TIMP1 ratios were significantly higher in the SHR control diet groups than their WKY rat counterparts, suggesting that MMP2 and MMP9 activity was elevated in the hypertensive rats (Fig 4B and 4D).

### The effects of hydralazine administration and interactions with diet in NASH

We compared groups of hypertensive SHRs fed the HFC or control diet and with or without hydralazine-treated water. Hydralazine was an effective antihypertensive agent in these rats and significantly lowered blood pressure regardless of diet (Fig 1B).

**Fig 5:**
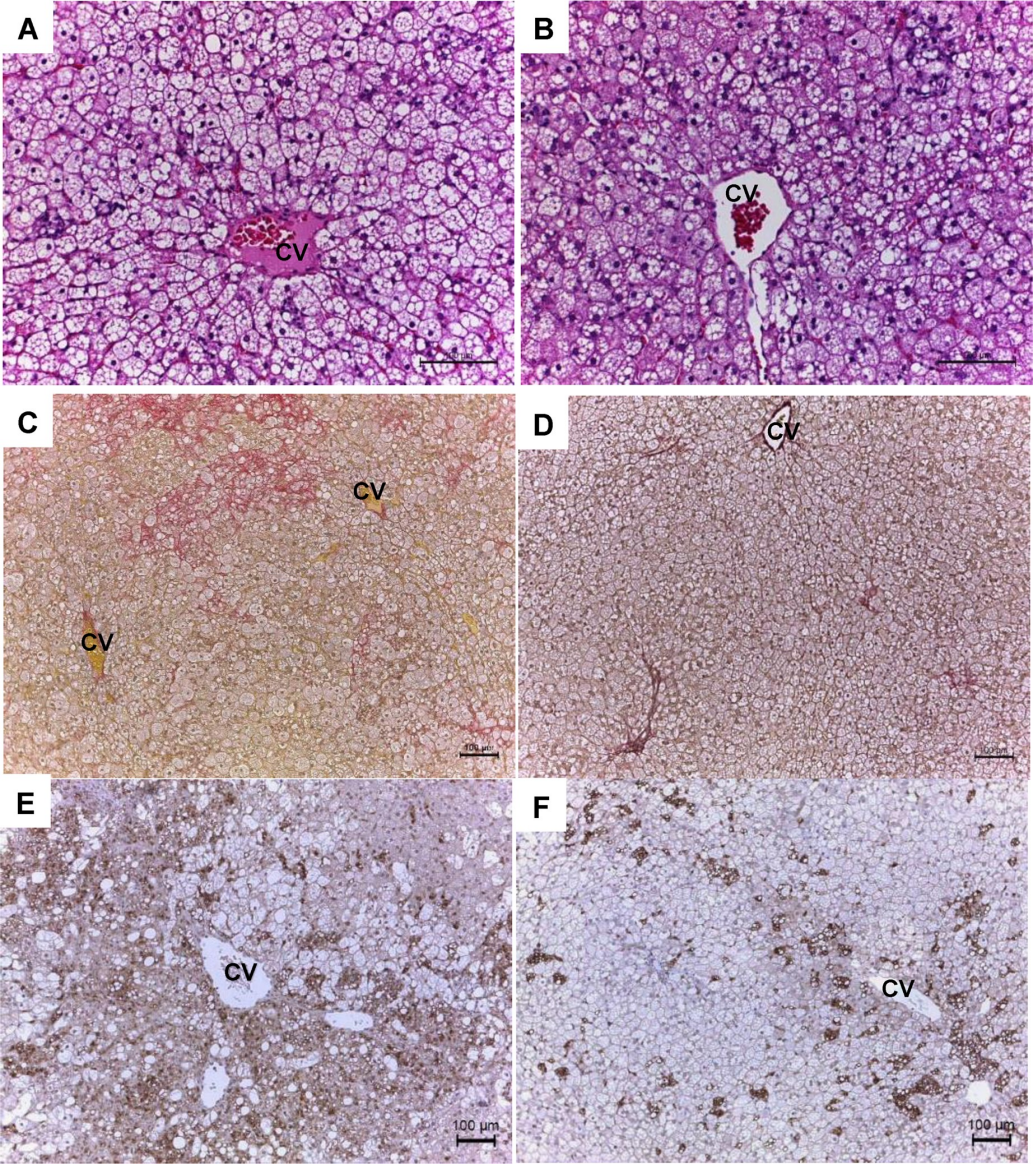
Images of liver sections in the second experiment. Representative images of liver sections subjected to H&E staining (A, B) [magnification: ×200], EVG staining (C, D) [magnification: ×100], and immunostaining with CD68 antibody (E, F) [magnification: ×100]. Liver sections from SHRs fed HFC diet in the absence (A, C, E) or the presence (B, D, F) of hydralazine. Scale bar, 100 μm. CV, central vein.

H&E staining was performed to evaluate the effects of hydralazine on the HFC diet-induced hepatic histopathology. Steatosis and hepatocellular degeneration were not observed in the liver of SHRs fed control diet in the presence or absence of hydralazine (data not shown). As seen in Fig 5A, macrovesicular steatosis, hepatocyte ballooning, and inflammatory cell infiltration were noted in the HFC-fed SHRs in the absence of hydralazine. Meanwhile, hydralazine treatment led to moderate alleviation of hepatocyte degeneration (Fig 5B).

**Fig 6:**
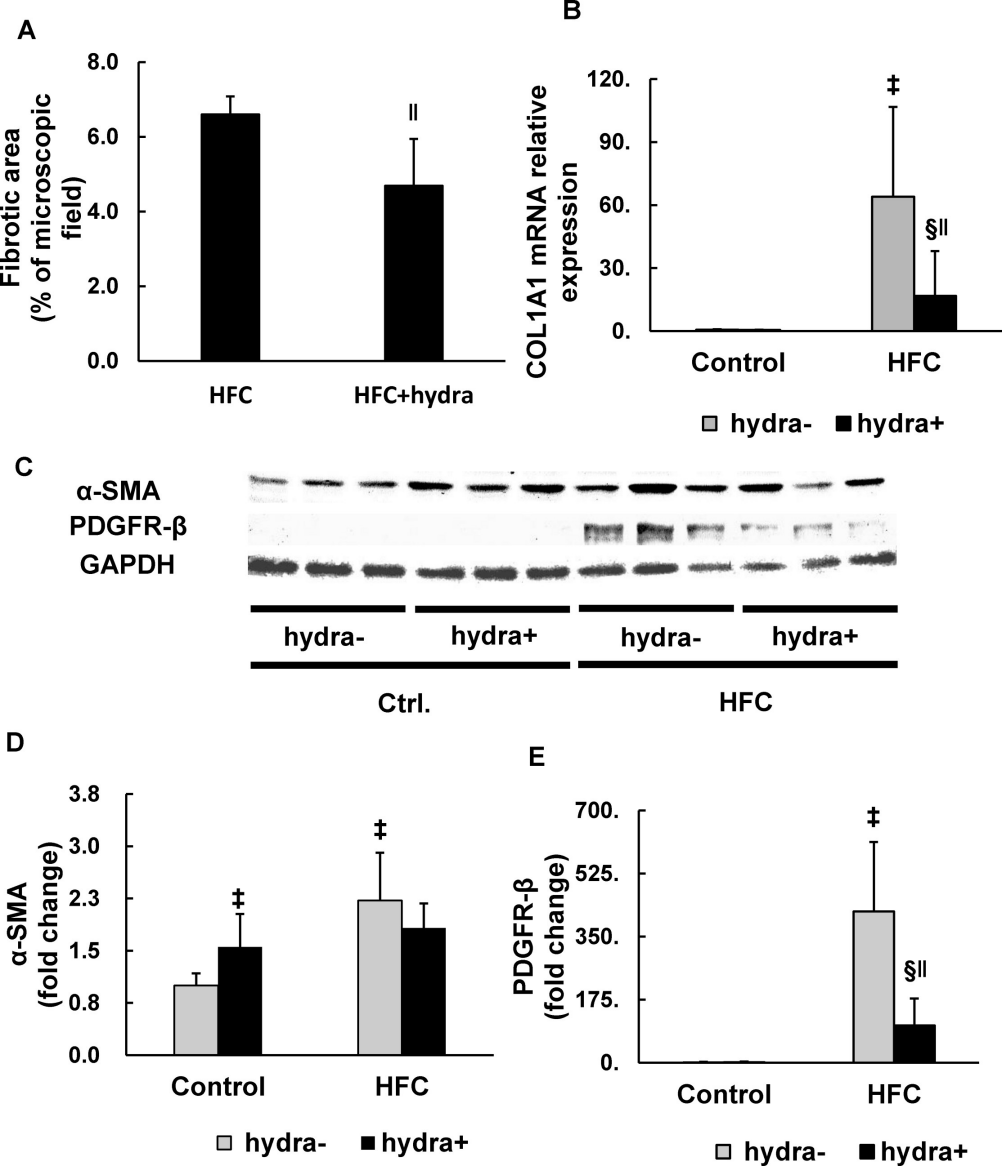
Fibrotic area and inhibitory effects of hydralazine on HFC-induced changes in the expression of fibrosis-related genes in the liver of SHRs. The quantification of fibrotic areas (%) in the EVG-stained liver sections (A). Hepatic expression of COL1A1 mRNA (B). Western blots of the hepatic expression of α-SMA (C, D) and PDGFR-β (C, E). n = 6/group. Significant interaction between hydralazine treatment and HFC diet was observed in the levels of COL1A1 mRNA, α-SMA, and PDGFR-β proteins. ^‡^P < 0.05 vs control diet group (one-way ANOVA); ^§^P < 0.05 vs control diet with hydralazine treatment (one-way ANOVA); ^‖^P < 0.05 vs HFC diet group (one-way ANOVA). COL1A1, collagen, type I, alpha-1 chain; α-SMA, alpha-smooth muscle actin; PDGFR-β, platelet-derived growth factor receptor-β.

EVG staining was also performed to evaluate liver fibrosis (Fig 5C and 5D), and the fibrotic area was quantified (Fig 6A). Hepatic fibrosis did not occur in SHRs fed control diet, whereas it was observed in the rats fed the HFC diet in the absence of hydralazine (Fig 5C). Hydralazine significantly decreased EVG-visualized, HFC-induced liver fibrosis (Figs 5D and 6A). The HFC diet resulted in intense CD68 immunolabeling in liver sections (Fig 5E), whereas hydralazine markedly reduced it (Fig 5F).

We also investigated the effects of hydralazine on body and liver weight as well as liver function in SHRs (Table 1). Significant interaction between the HFC diet and hydralazine treatment was observed in body weight, liver:body weight ratio, serum levels of insulin, and AST, ALT, and GGT levels. The HFC diet decreased body weight but increased liver weight and the ratio of liver to body weight. Hydralazine treatment suppressed HFC-induced increase in the liver:body ratio. Furthermore, hydralazine treatment significantly suppressed the HFC-induced increase in AST, ALT, and GGT levels; however, it increased the serum level of AST but not ALT and GGT in SHRs fed control diet. These results suggested that hydralazine markedly ameliorated liver damage led by the HFC diet; however, it might have caused a certain degree of hepatic injury in SHRs before HFC feeding. Serum TG and AST values of control group were slightly different from those of previous experiment [8]. This is probably because animals of the previous experiment were fasted before dissection, but not in the present one.

**Table 1:** The effects of hydralazine on body and liver weights as well as the levels of various biochemical indices in serum and liver.

	Ctrl.	Ctrl.+Hydra	HFC	HFC+Hydra
**Body weight (g)**	356 ± 9.1	344 ± 4.5^‡^	315 ± 12.9^‡^	328 ± 17.9
**Liver weight (g)**	13.4 ± 0.4	13.0 ± 0.3	28.1 ± 2.2*	26.8 ± 1.6*
**Liver/body weight (%)**	3.8 ± 0.1	3.8 ± 0.1	8.9 ± 0.4^‡^	8.2 ± 0.6^§^^‖^
**Serum**				
**Glucose (mg/dl)**	193 ± 12.0	195 ± 16.4	138 ± 7.6*	153 ± 9.4*
**Insulin (ng/ml)**	15 ± 4.2	18 ± 4.1	29 ± 4.8^‡^	21 ± 6.1^‖^
**TG (mg/dL)**	80 ± 10.7	58 ± 19.8^†^	33 ± 7.4*	31 ± 13.2*^†^
**TC (mg/dL)**	77 ± 2.3	74 ± 5.3^†^	188 ± 19.0*	160 ± 17.7*^†^
**AST (IU/L)**	88 ± 6.6	120 ± 11.8^‡^	286 ± 52.0^‡^	223 ± 34.1^§^^‖^
**ALT (IU/L)**	55 ± 4.8	57 ± 10.6	128 ± 22.6^‡^	94 ± 16.6^§^^‖^
**GGT (IU/L)**	1.5	1.5	4.8 ± 1.5^‡^	
**Liver**				
**TG (mg/g)**	21.5 ± 7.2	8.2 ± 4.5^†^	30.5 ± 11.6*	27.8 ± 7.6*^†^	2.6 ± 1.2^§^^‖^
**TC (mg/g)**	1.9 ± 0.7	2.0 ± 0.5	107.8 ± 11.5*	98.4 ± 26.6*

Data represent the mean ± SD (*n* = 6)

*P < 0.05 between control diet group and HFC diet group with two-way ANOVA.

^†^P < 0.05 between non hydralazine group and hydralazine group with two-way ANOVA.

^‡^P < 0.05 vs control diet group with one-way ANOVA.

^§^*P* < 0.05 vs control diet + hydralazine group with one-way ANOVA.

‖P < 0.05 vs HFC diet group with one-way ANOVA. TG: Triglyceride; TC: Total cholesterol; AST: Spartate aminotransferase; ALT: Alanine aminotransferase; GGT: Gamma-glutamyl transferase.

NAFLD/NASH is usually accompanied by insulin resistance [3]. Here, we investigated the effect of hydralazine on insulin resistance by evaluating the serum levels of glucose and insulin in SHRs. Hydralazine treatment tended to raise the glucose level, which was decreased in the HFC-fed rats. The HFC diet induced a higher serum level of insulin in SHRs, whereas hydralazine decreased it in the HFC-fed rats. Therefore, these findings suggest that hydralazine moderately ameliorates insulin resistance in SHRs.

Impaired lipid metabolism is often noted in patients with chronic liver diseases, and lipid profile has been suggested to be associated with the severity of liver damage [25]. Therefore, the levels of TG and TC in the serum and liver were evaluated to investigate the effect of hydralazine on lipid metabolism. The HFC diet significantly induced the increase in serum and hepatic TC and hepatic TG as well as the decrease in serum TG. Hydralazine suppressed the HFC-induced increase in hepatic TG as well as serum TC levels. These findings suggest that hydralazine ameliorated HFC-induced ectopic lipid metabolism. Conversely, it decreased the serum and hepatic TG levels as well as the serum TC levels in SHRs fed control diet.

### The effect of hydralazine on the expression of fibrosis-related genes

To investigate the effect of hydralazine on HFC-induced hepatic fibrogenesis, the expression of fibrosis-related genes in the liver, including COL1A1, α-SMA, and PDGFR-β, was evaluated (Fig 6B–6E). Significant interaction between the HFC diet and hydralazine was observed in the levels of COL1A1 mRNA, α-SMA, and PDGFR-β protein. Hydralazine did not affect the mRNA expression of COL1A1 in the control group of SHRs, whereas it markedly suppressed the HFC-induced upregulation of COL1A1 (Fig 6B). Hydralazine moderately increased the α-SMA level in the control group, whereas it did not affect the HFC diet-induced elevation of hepatic α-SMA in SHRs (Fig 6D). It did not change the hepatic level of PDGFR-β protein in the control group; however, it significantly suppressed the HFC-induced increase in the protein level (Fig 6E).

### The effect of hydralazine on pro-inflammatory and profibrotic cytokines

**Fig 7:**
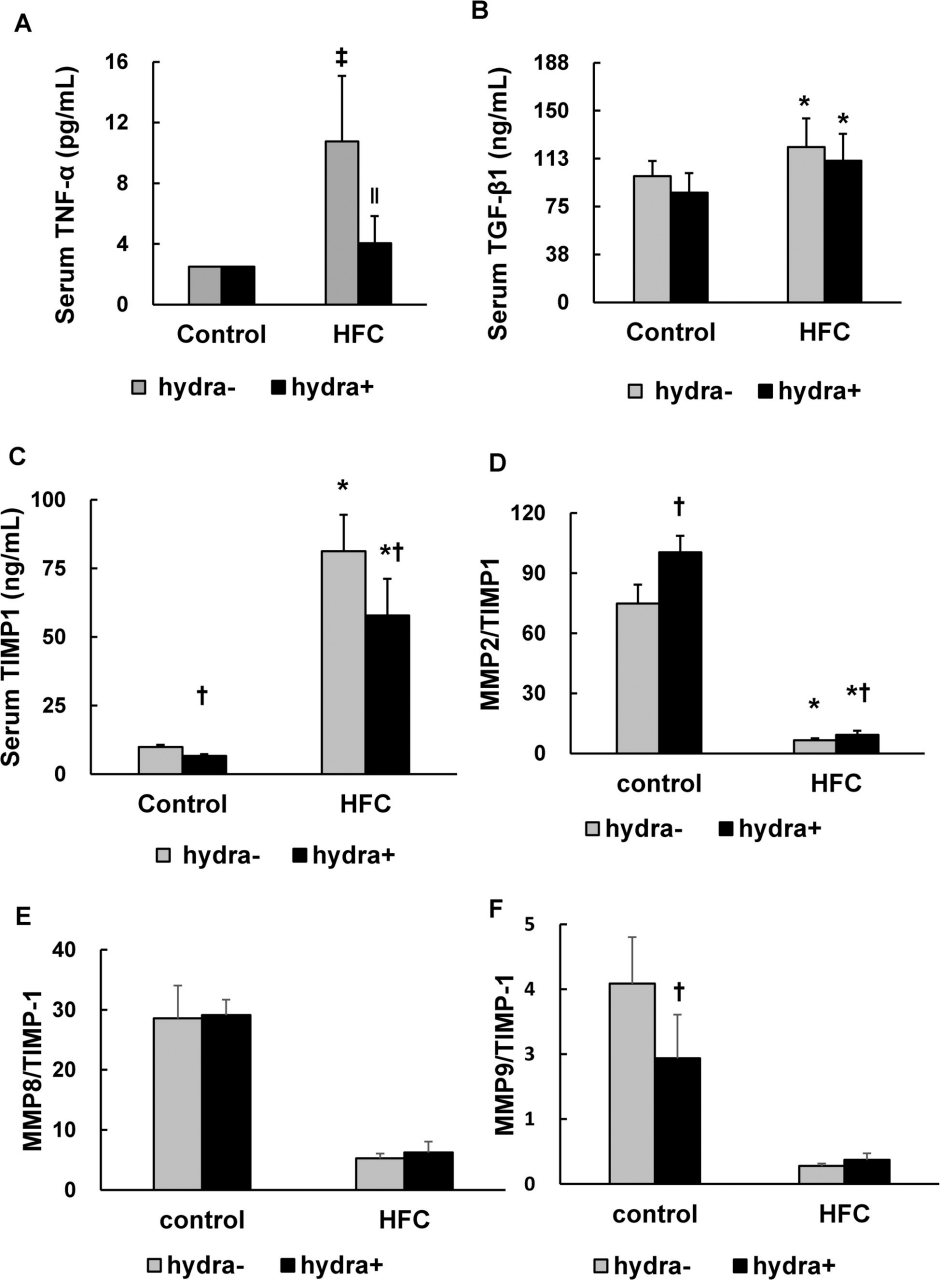
Suppressing effects of hydralazine on HFC-induced changes in the serum levels of pro-inflammatory and pro-fibrotic cytokines and ratios of MMPs to TIMP1 in SHRs. Serum levels of TNF-α (A), TGF-β1 (B), TIMP1 (C), MMP2 (D, MMP8 (E) and MMP9 (F). The ratio of MMP2 to TIMP1 as a proxy for MMP2 activity. n = 6/group. Significant interaction between hydralazine treatment and HFC diet was observed in the levels of serum TNF-α. *P < 0.05 between control diet and HFC diet groups (two-way ANOVA); †P < 0.05 between with and without hydralazine groups (two-way ANOVA); ‡P < 0.05 vs control diet group (one-way ANOVA); ‖P < 0.05 vs HFC diet group (one-way ANOVA). Abbreviations: TNF-α, tumor necrosis factor-alpha; TGF-β1, transforming growth factor-beta 1; TIMP1, tissue inhibitor of metalloproteinase-1; MMP, matrix metalloproteinases.

We investigated the effect of hydralazine on TNF-α and TGF-β1 expression by evaluating their serum levels in SHRs. Significant interaction between the HFC diet and hydralazine was observed in the level of serum TNF-α but not in that of TGF-β1. Hydralazine did not affect TNF-α levels in the control group but significantly suppressed the HFC-induced increase in TNF-α levels (Fig 7A). Hydralazine did not significantly affect the elevation of serum TGF-β1 in SHRs with the HFC diet (Fig 7B).

### The effect of hydralazine on MMP expression and activity

Hydralazine slightly reduced the level of TIMP1 in the control diet group and significantly suppressed the HFC diet-induced increase in TIMP1 expression (Fig 7C). It increased the ratio of MMP2 to TIMP1 expression in SHRs fed the control diet as well as the HFC diet (Fig 7D). Hydralazine treatment did not affect the MMP8:TIMP1 and MMP9:TIMP1 ratios regardless of diet (Fig 7E and 7F).

## Discussion

**Fig 8:**
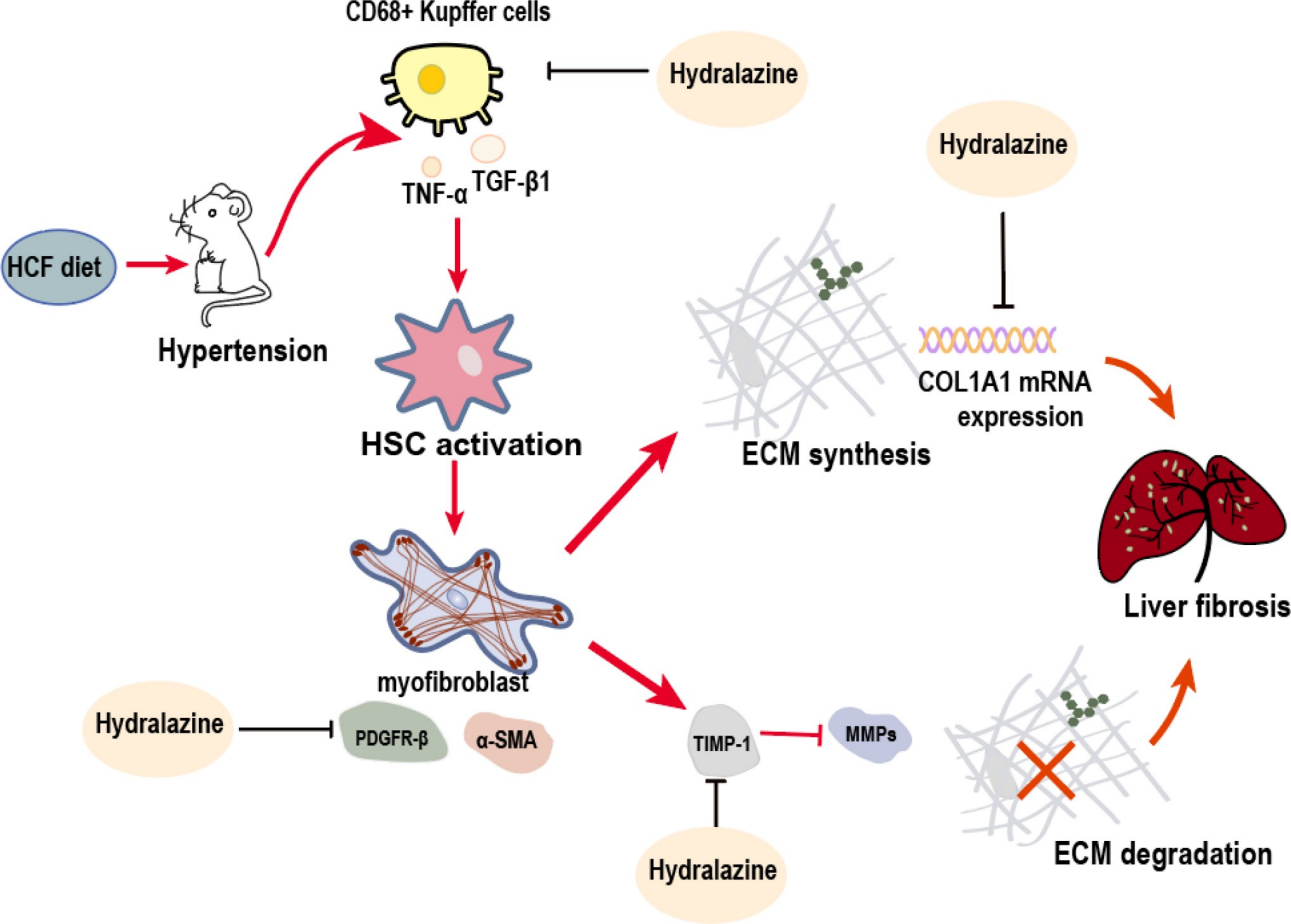
The possible mechanism underlying hepatic fibrogenesis during the progression of hypertension-associated NASH [9,10,14] and inhibition by hydralazine. The combined action of hypertension and HFC diet induces increased aggregation of CD68-positive Kupffer cells and results in the elevation of both serum TGF-β1 and TNF-α, the cytokines involved in liver inflammation and fibrosis. These cytokines, derived from Kupffer cells or other types of liver cells, induce increased activation of HSCs, which then differentiate into fibrogenic myofibroblasts and produce the major components of ECM (such as collagen), indicated by elevated upregulation of α-SMA and PDGFR-β. Meanwhile, myofibroblasts also participate in TIMP1 expression, the inhibitor of MMPs. HFC diet induced greater increase in serum TIMP1 as well as a greater decrease in MMP activities in hypertensive context compared with the ones under normotensive conditions. Since increased collagen synthesis was not noted in the hypertensive context, hypertension mainly enhanced the effects of HFC diet on ECM degradation and further resulted in more severe liver fibrosis. On the other hand, hydralazine, the antihypertensive agent, significantly attenuates the progression of HFC-induced liver fibrosis under hypertensive conditions by suppressing the aggregation of Kupffer cells and the elevation of serum TNF-α. It also reduces HFC-induced increases in the hepatic expression of PDGFR-β protein and COL1A1 mRNA, suggesting that hydralazine suppresses HFC-induced ECM synthesis. Furthermore, hydralazine significantly suppresses HFC-induced elevation of serum TIMP1, whereas its effects on the levels of MMPs are not prominent. Therefore, the effect of hydralazine on ECM degradation is still unclear. In conclusion, hypertension enhances HFC-induced hepatic fibrogenesis through increasing the suppression of MMP-mediated ECM degradation, whereas hydralazine attenuates liver fibrosis development mainly by suppressing HFC-induced ECM synthesis under hypertensive conditions.

Here we determined that hepatic fibrosis induced by the HFC diet was exacerbated in a hypertensive environment (the SHR model) due, at least in part, to reduced MMP activity and the concomitantly reduced degradation of the ECM. The antihypertensive agent hydralazine significantly attenuated the progression of liver fibrosis; based on our results, suppression of ECM synthesis was the likely underlying mechanism (Fig 8).

First, we compared the development of HFC-induced hepatic fibrosis between hypertensive SHRs and the normotensive WKY strain. In SHRs, the HFC-induced increase in CD68-positive liver macrophages was exacerbated. The activation and proliferation of HSCs, the major source of ECM components [14,26], were also enhanced following HFC feeding in SHRs, as indicated by increased upregulation of the α-SMA protein. PDGFR-β proteins and COL1A1 mRNA upregulation was similar between strains, suggesting that the differences in ECM deposition were related to its degradation and not its synthesis. This is supported by a greater elevation of serum TIMP1 as well as a greater reduction in MMP activity (MMP2, MMP8 and MMP9) in SHRs which would reduce ECM degradation. When SHRs were treated with the antihypertensive agent hydralazine, HFC diet-induced hepatic fibrosis was significantly reduced. Given its attenuation of inflammatory processes (macrophage aggregation and TNF-α release) and reduction of fibrosis-related gene expression (PDGFR-β protein), hydralazine suppressed the HFC-induced activation and proliferation of HSCs as well as reduced collagen I (ECM) synthesis. However, hydralazine’s effects on ECM degradation are less clear.

Leroux et al. revealed that a dysregulation of lipid metabolism in mice fed a high-fat diet induced toxic lipid accumulation in Kupffer cells and primed them to recruit lymphocytes and exhibit a pro-inflammatory phenotype [27]. Our previous study showed that compared with the normotensive WKY rats, the hypertensive strains (SHR and SHRSP5/Dmcr) fed with the control diet had higher serum TG and lower serum TC levels, suggesting that dysregulation of lipid metabolism occurs in hypertensive strains before HFC feeding. Meanwhile, the HFC diet induced a greater increase in the serum TC levels in the hypertensive rats, suggesting that HFC-induced disturbance in lipid metabolism is enhanced in the hypertensive context. Therefore, the lipid metabolism abnormity might be responsible for the increased accumulation of hepatic macrophages in hypertensive SHRs. Our previous study also showed that both serum TNF-α and TGF-β1 levels were elevated in SHRs fed the HFC diet, whereas only TNF-α was increased in the normotensive WKY strain [8]. The HFC diet induced a greater activation of the inflammatory signals of TGF-β1/MAPK and suppressed anti-oxidative and anti-inflammatory Nrf2 signals. Therefore, increased activation of Kupffer cells as well as the increased release of the inflammatory cytokines was likely responsible for the more severe inflammatory response in hypertensive SHRs. Conversely, hydralazine treatment significantly attenuated the HFC-induced release of TNF-α and not just macrophage accumulation in SHRs. Overexpression of TNF-α is considered a hallmark of NAFLD-related inflammation; meanwhile, it is also associated with insulin resistance [12]. Barbuio et al. determined that the inhibition of TNF-α by infliximab, a potent TNF-α neutralizing monoclonal antibody, resulted in amelioration of hepatic inflammation and fibrosis as well as improved insulin signal transduction in Wistar rats fed a high-fat diet [28]. Here, hydralazine treatment moderately suppressed the HFC-induced increase in serum insulin and might have counteracted the HFC-induced changes in serum glucose in SHRs. Therefore, hydralazine treatment was believed to have ameliorated hepatic inflammation and insulin resistance in hypertensive SHRs.

Hepatic fibrosis results from either increased synthesis or decreased degradation of ECM. Fibrogenic mediators activate HSCs, the major ECM-producing cells in injured liver [29], to increase ECM synthesis or to reduce degradative MMP activity. In hypertensive SHRs fed the HFC diet, we saw higher increases in hepatic α-SMA levels (a marker for HSC activation) than those in the normotensive rats. Unexpectedly, the HFC diet induced COL1A1 similarly across strains, suggesting that hypertension did not further increase ECM synthesis. However, hydralazine suppressed the HFC diet-induced PDGFR-β upregulation as well as an increase in COL1A1 mRNA expression and may have restricted the increase in α-SMA. These results suggested that hydralazine reduced or prevented further fibrosis by suppressing HFC diet-induced activation of HSCs and their production of collagen I. The other mechanism—the decrease in MMP activity—is mainly due to the overexpression of their endogenous inhibitors (TIMPs) [14]. TIMP1 has been reported to be upregulated in serum and liver during the progression of hepatic fibrosis in human as well as animal models [30–32]. Kasahara et al. determined that serum TIMP1 was positively correlated with the degree of liver fibrosis in patients with chronic hepatitis C [31], whereas Prystupa et al. showed that the activities of MMP2, MMP8, and MMP9 in serum might be markers of progression of human alcoholic liver disease [16]. Here, we determined that the HFC diet exacerbated serum TIMP1 elevation and, thus, further reduced MMP (MMP2, MMP8, and MMP9) activity in hypertensive SHRs when compared with that in the normotensive WKY strain. Hydralazine treatment significantly suppressed the HFC-induced increase in serum TIMP1; however, it only weakly increased MMP2 activity and did not affect MMP8 and MMP9 activity in hypertensive SHRs. Thus, our data indicate that the HFC diet induced increased fibrosis in a hypertensive context by overexpression of TIMP1 and reduction of MMP activity, whereas hydralazine administration attenuated the fibrosis primarily by suppressing collagen I synthesis. In addition, we noted differences in hepatic α-SMA proteins between strains fed the control diet as well as higher activities of MMP2 and MMP9 in the hypertensive strain, suggesting that the balance of HSC-mediated ECM synthesis and degradation was impaired in the hypertensive rats separately from any HFC diet-induced disturbances. Furthermore, the inflammatory system might also be imbalanced independently of the HFC diet. Our previous study found reduced pro-inflammatory (nuclear factor-κB) signaling and increased anti-inflammatory [Nrf2/Kelch-like ECH-associated protein 1] signaling in SHRs fed the control diet, suggesting that dysregulation of the inflammatory system was present [8] in the hypertensive strain.

Hydralazine is a direct arteriolar vasodilator, and its vasodilatory effects were achieved by altering intracellular calcium release and disturbing the calcium influx in smooth muscle cells, which results in the modification of the contractile state of the arterial vascular smooth muscle [33]. In the present study, HFC feeding appeared to result in an insufficient blood supply to the edge of the livers in hypertensive SHRs, which might initiate the process of hepatic fibrosis. Therefore, we selected hydralazine as the therapeutic agent. In the study conducted by Kozono et al., a high-salt diet was administered to induce severe hypertension, whereas a choline-deficient, L-amino acid-defined diet was administered to induce steatohepatitis in SHRs [34]. They showed that antihypertensitve therapy with amlodipine or hydralazine improved insulin resistance and imbalances in the expression of immunological factors. Consistent with their findings, we also showed that hydralazine treatment improved insulin resistance. Additionally, hydralazine treatment partially reversed the effect of hypertension on the development of hepatic fibrosis. Therefore, our results suggest that antihypertensive therapy with hydralazine significantly attenuated HFC diet-induced hepatic fibrosis in hypertensive SHRs by decreasing the blood pressure as well as improving insulin resistance.

In conclusion, our study suggests that hypertension provides a pathological environment that induces mild liver damage and exacerbates the HFC diet-induced inflammatory response and liver fibrosis in NASH by altering the ECM synthesis–degradation balance. The antihypertensive hydralazine ameliorated HFC-induced hepatic fibrosis by lowering blood pressure and improving insulin resistance.

## Supporting information

S1 File (PDF)
