# Supplementary material for: The antihypertensive agent hydralazine reduced extracellular matrix synthesis and liver fibrosis in nonalcoholic steatohepatitis exacerbated by hypertension
Source: PLoS One. 2020 Dec 14;15(12):e0243846. doi: 10.1371/journal.pone.0243846 (PMC7735612; doi:10.1371/journal.pone.0243846)
Supplement: S1 File — (PDF) [file pone.0243846.s001.pdf]

Raw data for this page

1st experiment (WKY, SHR)

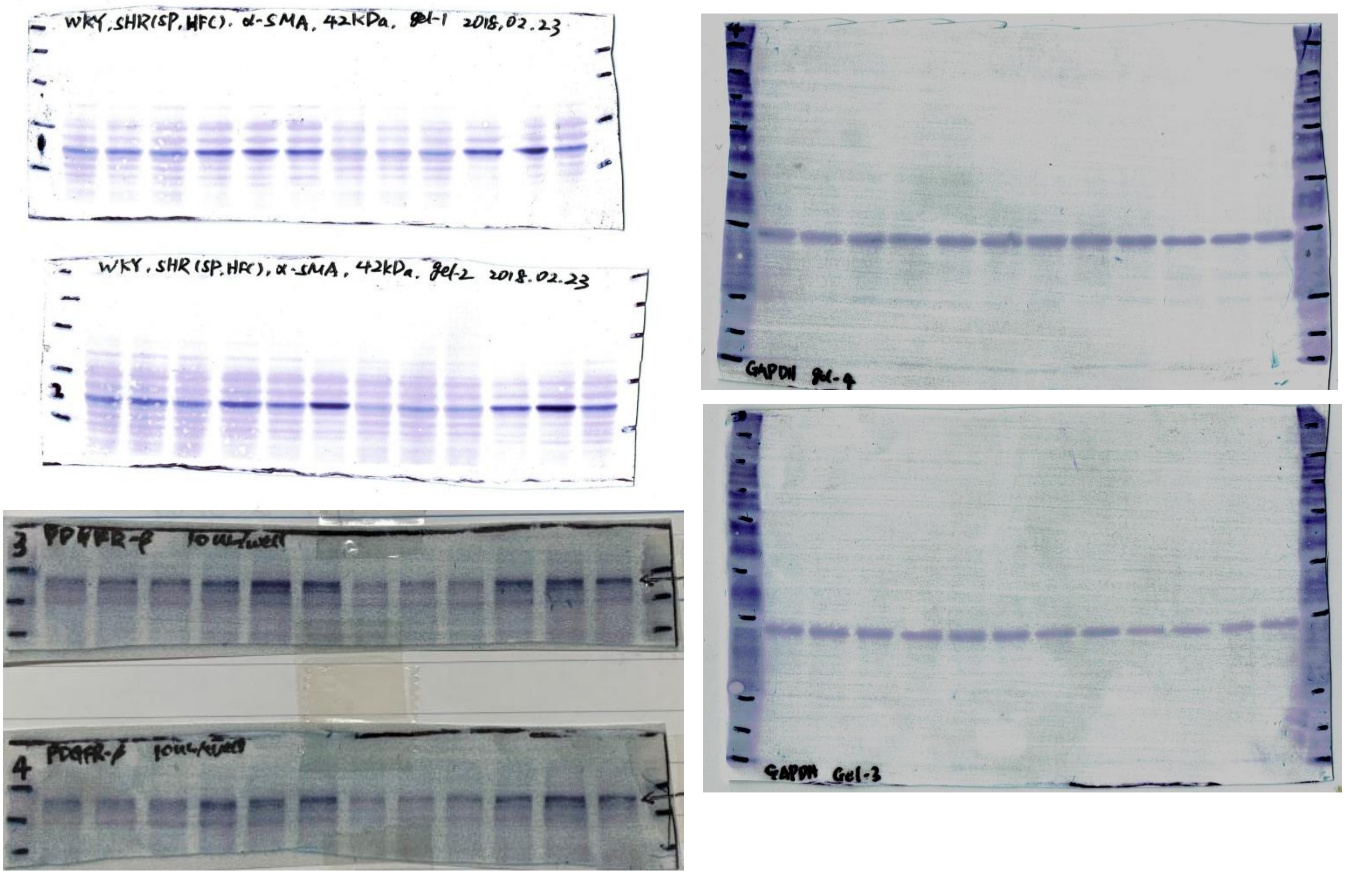

WKY Ctrl. HFC SHR Ctrl. HFC

	No.	WKY-SP	WKY-HFC	SHR-SP	SHR-HFC
COL1A1/GAPDH mRNA	1	1	14.1087	2.5526	9.8103
	2	0.7507	8.0876	1.8320	125.9915
	3	0.9182	24.8737	1.5387	31.5550
	4	0.7088	29.7973	1.4364	24.3990
	5	0.8526	6.0297	1.6635	5.1776
	6	0.9373	12.4911	2.5952	8.4211
TBARS	1	0.7542	0.8441	0.2379	2.4829
	2	0.8738	0.9438	0.9438	1.4811
	3	0.9438	1.5512	0.5701	1.8549
	4	0.7336	0.9906	1.2943	1.6213
	5	2.2053	1.1774	0.9438	2.4156
	6	1.22425	0.5934	none	1.9250
Serum TIMP-1	1	9.76	31.52	9.91	76.67
	2	10.98	70.30	9.42	112.20
	3	8.90	58.53	10.22	109.77

	4	10.51	38.64	9.51	89.30
	5	9.99	46.00	9.14	92.07
	6	10.51	38.51	10.26	66.72
Serum MMP2	1	432.15	501.52	651.16	671.94
	2	370.06	543.83	697.84	695.25
	3	442.87	733.98	700.42	682.31
	4	413.33	538.56	713.34	998.58
	5	488.24	514.77	726.24	859.43
	6	567.52	509.47	575.40	674.53
Serum MMP8	1	331.30	481.81	328.07	483.04
	2	299.87	799.36	227.71	714.73
	3	337.75	603.29	256.51	636.13
	4	253.30	445.98	238.49	430.93
	5	244.89	517.32	316.38	555.34
	6	323.23	579.10	326.86	484.26
Serum MMP9	1	22.46	38.16	52.53	66.62
	2	30.31	90.07	36.10	70.28
	3	40.34	67.53	42.27	77.35
	4	26.25	45.65	39.04	64.33
	5	24.68	85.21	30.56	67.77
	6	32.65	47.01	46.45	62.27

Not all orders(No.)match except for serum MMPs and TIMP-1.

2nd experiment (SHR)

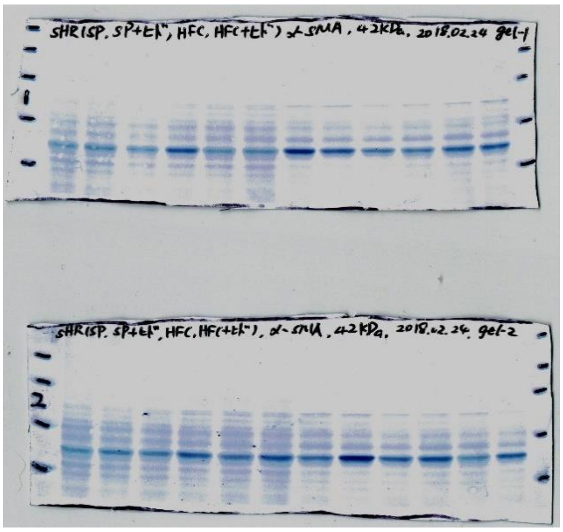

SHR Ctrl. Ctrl.+hy HFC HFC+hy

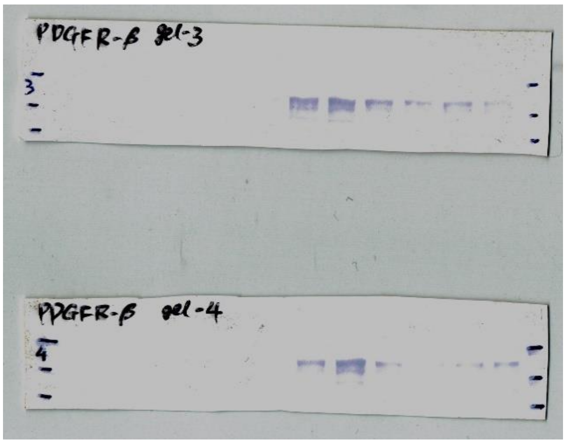

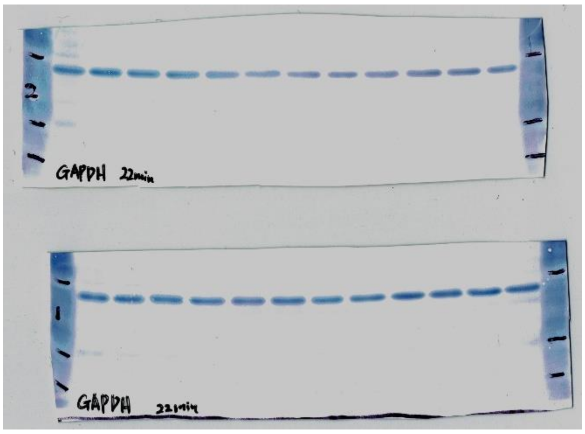

	No	Ctrl	Ctrl+Hydra	HFC	HFC+Hydra
bodyweight	1	341	344	296	342
	2	364	344	322	333
	3	356	337	316	314
	4	351	360	319	355
	5	363	350	333	312
	6	363	346	304	314
liver weight	1	13.123	13.031	25.551	27.57
	2	14.039	12.602	31.004	24.368
	3	13.158	12.771	27.041	25.174
	4	13.33	13.326	27.053	28.296
	5	13.782	13.304	30.565	27.68
	6	13.196	12.762	27.489	27.646
Glucose	1	193	188	147	145
	2	211	204	134	158
	3	204	222	145	141
	4	182	187	141	167
	5	182	191	134	151
	6	187	175	127	156
Insulin	1	10.12	18.18	33.53	18.78
	2	12.67	21.87	29.65	18.31
	3	12.20	18.52	30.68	15.91
	4	21.58	22.62	19.89	18.52
	5	17.56	11.07	29.45	24.27
	6	15.92	18.56	32.15	32.55
TG	1	88	98	47	22
	2	76	50	25	36
	3	82	54	31	32
	4	80	52	33	55
	5	93	47	32	23
	6	62	47	30	20
TC	1	78	79	177	209
	2	78	75	178	337
	3	75	80	188	255
	4	81	69	165	265
	5	77	67	215	310
	6	75	72	206	341
AST	1	96	120	134	195
	2	85	140	150	240
	3	94	123	172	206
	4	78	112	152	183

	5	88	118	183	239
	6	85	105	167	274
ALT	1	50	50	112	92
	2	49	58	167	99
	3	55	78	106	92
	4	54	51	123	80
	5	62	55	118	78
	6	57	51	141	124
GGT	1	1.5	1.5	3	1.5
	2	1.5	1.5	5	1.5
	3	1.5	1.5	4	3
	4	1.5	1.5	4	1.5
	5	1.5	1.5	6	4
	6	1.5	1.5	7	4
Liver TG	1	21	1.9	31.2	15.4
	2	16.3	5.8	51.8	25.6
	3	17.3	9.5	30.8	31.2
	4	27.1	6.4	28.3	30.7
	5	32.8	15	22	38.1
	6	14.2	10.7	18.7	26.1
Liver TC	1	1.65	1.77	64.2	99.5
	2	1.17	1.85	74.1	116
	3	2.17	1.77	129.1	101.9
	4	2.97	2.97	126.6	101.4
	5	1.41	1.45	102.8	127.6
	6	2.13	2.29	93.7	100.5
COL1A1/GAPDH mRNA	1	1	0.4819	105.2256	6.3790
	2	0.4676	0.3364	17.3288	5.9361
	3	0.5575	0.3508	120.1973	4.2505
	4	0.5017	0.7771	28.6751	59.6896
	5	0.5783	0.5554	75.5668	13.1568
	6	none	0.534486717	36.9720	11.2211
Serum TNF α	1	2.5	2.5	6.247	2.108
	2	2.5	2.5	13.509	2.045
	3	2.5	2.5	13.580	5.572
	4	2.5	2.5	14.010	6.451
	5	2.5	2.5	4.239	4.239
	6	2.5	2.5	12.938	3.845
Serum TGF-β1	1	105.15	73.91	124.85	108.46
	2	119.05	99.95	156.99	150.44
	3	94.13	86.65	105.69	101.12

	4	87.28	61.83	106.65	89.70
	5	98.47	96.88	136.85	114.12
	6	88.96	96.45	98.68	101.12
Serum TIMP-1	1	10.35	7.22	100.75	48.49
	2	10.86	6.65	75.13	56.33
	3	10.06	6.68	81.12	70.48
	4	9.74	7.45	87.64	73.92
	5	10.14	6.00	82.34	59.58
	6	8.50	5.99	60.78	38.47
Serum MMP2	1	705.15	640.21	506.22	566.37
	2	738.42	652.29	468.69	554.33
	3	747.50	682.49	575.40	509.22
	4	788.38	838.39	530.26	492.70
	5	679.47	631.16	578.41	524.25
	6	771.72	575.40	480.70	437.22
Serum MMP8	1	326.86	205.78	476.92	389.13
	2	328.07	199.80	302.28	499.76
	3	227.71	165.28	514.46	355.93
	4	238.49	242.89	510.78	316.38
	5	256.51	178.75	441.09	343.81
	6	316.38	173.99	320.81	215.74
Serum MMP9	1	46.17	12.75	28.89	22.03
	2	58.61	10.58	29.91	31.18
	3	31.67	26.23	27.66	24.67
	4	33.25	20.08	28.44	26.11
	5	36.18	13.26	33.66	25.04
	6	26.80	14.02	19.58	25.70
Blood Pressure 8w	1	186	182	188	179
	2	180	185	186	183
	3	181	180	185	174
	4	185	184	174	179
	5	182	195	179	185
	6	171	170	181	173
Blood Pressure 9w	1	191	159	184	161
	2	185	163	202	157
	3	172	164	204	155
	4	180	166	190	178
	5	181	165	186	155
	6	191	167	179	156
Blood Pressure 10w	1	194	158	196	161
	2	216	156	189	167

	3	196	159	197	163
	4	195	165	198	162
	5	191	161	196	155
	6	199	155	187	146
Blood Pressure 12w	1	179	153	198	146
	2	217	150	186	146
	3	191	154	186	146
	4	219	163	176	154
	5	205	153	199	130
	6	210	147	191	123
Blood Pressure 14w	1	213	167	186	149
	2	205	168	193	127
	3	203	153	181	158
	4	213	158	184	152
	5	200	145	169	123
	6	211	150	183	136
Blood Pressure 16w	1	206	156	197	142
	2	217	162	191	147
	3	191	165	173	154
	4	211	156	187	153
	5	224	152	188	132
	6	214	156	177	147
Blood Pressure 18w	1	218	146	204	145
	2	214	173	187	153
	3	225	165	184	173
	4	235	167	174	160
	5	231	135	180	144
	6	227	166	199	148

Not all orders(No.)match except for serum MMPs/TIMP-1.
